# Genetic Variations of *RAD51* and *XRCC2* Genes Increase the Risk of Colorectal Cancer in Bangladeshi Population

**DOI:** 10.31557/APJCP.2020.21.5.1445

**Published:** 2020-05

**Authors:** Anika Uddin Hridy, Samia Shabnaz, M d Asaduzzaman, Mohammad Shahriar, Mohiuddin Ahmed Bhuiyan, Mohammad Safiqul Islam, S M Moazzem Hossen, Talha Bin Emran

**Affiliations:** 1 *Department of Pharmacy, University of Asia Pacific, 74/A, Green Road, Dhaka-1205, Bangladesh. *; 2 *Department of Pharmacy, Noakhali Science and Technology University, Sonapur, Noakhali-3814, Bangladesh. *; 3 *Department of Pharmacy, Faculty of Biological Science, University of Chittagong, Chittagong-4331, Bangladesh.*; 4 *Department of Pharmacy, BGC Trust University Bangladesh, Chandanaish, Chittagong-4381, Bangladesh. *

**Keywords:** Colorectal cancer, genetic polymorphism, PCR-RFLP, RAD51, XRCC2, Bangladeshi population

## Abstract

**Objectives:**

In case of Bangladeshi population, no report is observed till now showing the genetic variations of *RAD51* (*rs1801320*) and *XRCC2* (*rs3218536*) genes polymorphism having association with colorectal cancer risk. For this reason the aim of this study is to ascertain their interrelation with colorectal cancer occurrence in Bangladeshi population.

**Materials and Methods:**

A case control study was conducted where 200 colorectal cancer patients and 200 healthy volunteers were figured for this research using Polymerase Chain Reaction-Restriction Fragment Length Polymorphism (PCR-RFLP).

**Results:**

Here, in case of *RAD51* (*rs1801320*), G/C heterozygous genotype was found significant (p=0.037; OR=1.64; 95% CI=1.03 to 2.6). On the other hand, G/G genotype was not found statistically significant (p=0.423; OR=1.61; 95% CI=0.49 to 5.22) and significance was observed for GC+GG (p=0.030; OR=1.63; 95% CI=1.05 to 2.55). In case of XRCC2 (rs3218536), C/T heterozygous genotype was remarked statistically significant (p=0.033; OR=1.60; 95% CI=1.04 to 2.46). The T/T genotype was not recorded statistically significant (p=0.237; OR=1.65; 95% CI=0.72 to 3.76) but significance found for CT+TT (p=0.027; OR=1.61; 95% CI=1.05 to 2.45). Moreover, it is found that the risk factor of developing CRC is observed in G/C, C/T heterozygote and GC+GG, CT+TT (heterozygote+ mutant) in *RAD51 *(*rs1801320*) and* XRCC2* *(rs3218536*) respectively although no significance is observed in case of G/G and T/T mutant.

**Conclusions:**

So, the association of *RAD51* (*rs1801320*) and *XRCC2 *(*rs3218536)* genes polymorphism with colorectal cancer risk is observed in Bangladeshi population.

## Introduction

In Bangladesh, cancer can be said as one of the uppermost reasons of expansion of death and morbidity in the approaching years. According to International Agency for Research on Cancer, in 2030 the death rate will be 13% and it was 7.5% in 2005 which determines the proportion is appraised to be precisely twofold compared to 2005 (Hussain and Sullivan, 2013). Presumption says, by 2040 the figure will be expanded from 18.1 million to 29.5 million. Colorectal cancer is attributed as the most usual malignant tumours affecting the parts of alimentary canal appeared from large intestine due to unusual extension of cells having potential to invade several parts of human body (Jemal et al., 2010). Depending on the initiating point, these types of cancers can be said either as colon or rectal cancer. Colon cancer is noticed to be identified in two-third cases and remaining one-third as rectal cancer (Lynch and De’aChapelle, 2003). 75% of CRC can be remarked as sporadic. In tumour development, a part of modification in case of other genes can be observed and the occurrence of ultimate addition of supplementary malicious attributes such as intrusiveness of tissue and the ability to metastasize takes place (Tariq and Ghias, 2016).

Several risk factors related to CRC are individual study of adenomatous polyps, one’s own record of inflammatory bowel disease, genealogical account of colorectal cancer or adenomatous polyps and inherited genetic risk factors, coronary artery disease notably (Neugut et al., 1995). Familial Adenomatous Polyposis (FAP) as well as Hereditary Non-Polyposis Colorectal Cancer (HNPCC) also called Lynch syndrome are the most frequent inherited conditions and approximately 5 to 10% of CRCs are a sequel of accepted hereditary condition (Jackson et al., 2006). The probability of colorectal cancer is actively governed by diet where modification in nutritional habits might diminish up to 70% of CRC implication (Willett, 2005). High meat consumption, diets with high fat specifically animal fats has also been bundled in colorectal cancer continuation (Santarell et al., 2008). At adolescent period, several aspects like physical inactivity and corpulence, smoking, strong drink consumption are responsible for the starting of colorectal cancer. Evidence shows that continuing regular use of aspirin along with Non-Steroidal Anti-Inflammatory Drugs (NSAIDS) lessen the probability of CRC considerably (Cook et al., 2013). 5-fluorouracil (5-FU) capecitabine, oxaliplatin and irinotecan are utilized predominantly in chemotherapy for the patients of CRC. Targeted therapy is regarded as an advanced sector of drug evolution. It is engaged with targeted therapy which consequences from expanded apprehension of the molecular differences necessitated in cancer occurrence. Particular molecules engaged in tumor magnification and development are featured by these drugs having divergent and generally less serious after effects compared to traditional chemotherapy drugs (Jemal et al., 2010).

The protein, indispensable for reconstructing the damaged DNA is formed by instructions provided with RAD51 gene which plays a fundamental role in homologous recombinational repair. It is evident from innumerable studies that *RAD51* is up regulated in different cancers. Attachment of the DNA with* RAD51* protein takes place at the site of a break and encompassed in a protein case which is indispensable introductory step in the restoring operation. *RAD51* gene is an extremely protected well-designated DNA repair protein (Cousineau et al., 2005). The scheme of *RAD51* along with molecular regulation has not yet been completely confirmed (Gildemeister et al., 2009).

The location of *XRCC2 *(X-ray repair cross complementing 2) is 7q36.1 which is a member of the RAD51-related protein family. Chromosomal fragmentation, translocations and deletions are thought to be repaired by XRCC2. Additionally, this gene is found to be necessitated in the HR repair (HRR) pathway of DNA double-stranded breaks (DSBs). An association between *XRCC2* (*rs3218536*) single-nucleotide polymorphisms (SNPs) and cancer incidence risk are found to be the most common mutation observed by numerous studies (Lin et al., 2011). The increment of cancer risk is appeared due to two known epigenetic causes of* XRCC2* deficiency. One of them is the *XRCC2* exponent methylation and epigenetic repression of* XRCC2* by over-expression of EZH2 protein. Depletion of homologous recombinational repair and epigenetic repression of *RAD51* paralogues including *XRCC2* are accompanied by increased expression of *EZH2*. Over-expression of EZH2 protein between 40%-75% is found in breast cancer and EZH2 mRNA is up-adjusted, about 7.5-fold (Paulicova et al., 2013; Chang and Hung, 2012; Volkel et al., 2015).

As a probable element for breast, colorectal, lung, prostate, endometrial as well as gastric cancers both *RAD51* (*rs1801320*) and* XRCC2 (rs3218536*) genes have been perceived in different ancestral groups. A case-control study on Bangladeshi population was conducted recently where the high risk association between altered gene expression of *RAD51* (*rs1801320*) gene polymorphism and breast cancer development was detected. In different ethnicities,* RAD51* (*rs1801320*) and *XRCC2* (*rs3218536*) gene polymorphisms are certified to be a threat aspect of colorectal cancer and *RAD51* (*rs1801320*) gene polymorphism in breast cancer evolution in Bangladeshi population. A possibility may arise basing on a correlation between *RAD51* (*rs1801320*) and *XRCC2* (*rs3218536*) polymorphisms with colorectal cancer risk in Bangladeshi population. Moreover, no related investigation has been supervised concerning this feasible association in Bangladeshi populace. We, consequently, presume to find out that predictable relationship of RAD51 (rs1801320) and *XRCC2* *(rs3218536*) genetic polymorphisms with colorectal cancer risk in Bangladeshi citizenry for the very first time.

## Materials and Methods

*Study population*

A case control research was co-ordinated on 200 colorectal cancer patients and 200 healthy volunteers basing on age, weight and sex. Colorectal cancer patients were enlisted from Ahsania Mission Cancer and General Hospital and National Institute of Cancer Research and Hospital (NICRH), Dhaka, Bangladesh. After that, physical analysis of controls were picked by matching age and genetic history to colorectal cancer patients. The age of patients ranged from 13 years to 89 years (mean 51 years). Most of the patients are observed having tumour stages I, II and IV (mostly stage II). Age, sex, diet, weight, BMI, TNM staging, demographic characteristics, lifestyle factors data were stockpiled via interrogations by skilled attendants in presence of specialized doctors. The demographic characteristics of the patients and the controls are presented in Table 1 and the correlation of *RAD51* (*rs1801320*) and *XRCC2* (*rs3218536*) polymorphism with clinic pathological characteristics of patients are mentioned in Table 2. In case of both *RAD51 *(*rs1801320*) and *XRCC2* (*rs3218536*) polymorphisms, patients who have primary location of cancer in rectum are found to be more susceptible to have colorectal cancer as statistical significances are observed; RAD51 (OR= 1.7; 95% CI= 1.45 to 5.14) and *XRCC2* (OR= 0.21; 95% CI= 0.11 to 0.41) and in both cases the p values observed are less than 0.05. No other factors are found to be significantly associated with *RAD51* (*rs1801320*) and *XRCC2* (*rs3218536*) polymorphisms. Approval of the study protocol was done by the ethical committees of the respective hospitals and the research was executed according to the descriptions of Helsinki and its following editions (World Medical Association Declaration of Helsinki, 2013). An informed consent document was signed by each patient and control subject after briefing the motive of the study. The genotyping scrutiny was done in the laboratory of Biotechnology, Faculty of Pharmacy, The University of Asia Pacific, Dhaka-1205, Bangladesh.

*Genotyping*

Genomic DNA was separated from the blood specimens assembled from all patients by the help of DNA extraction mini kit, Favorgen, Taiwan. Genomic DNA amplification was executed using the predesigned forward and reverse primers. PCR was done by following the standard protocol mentioned in published method (Shabnaz et al., 2015). In case of both* RAD51 *(*rs1801320*) and *XRCC2 *(*rs3218536*), PCR-Restriction Fragment Length Polymorphism (RFLP) approach was applied to genotype. PCR product of 157 bp and 234 bp were obtained for *RAD51* (*rs1801320*) and *XRCC2* (*rs3218536*) SNPs respectively. PCR amplifications conditions are given in Table 3. Digestion of 157 bp PCR product of *RAD51* (*rs1801320*) was dispatched with BstNI (NEB, USA) by incubating at 60° C for 4 hours which produced two fragments 86 bp and 71 bp in case of G/G allele while for C/C allele it produced only one fragment of 157 bp. Restriction enzyme Hph1 (NEB, USA) (37° C overnight) bisected the T/T allele of *XRCC2 (rs3218536*) giving two fragments of 147 bp and 87 bp but didn’t divide the C/C allele that is only one fragment 234 bp was observed. These DNA fragments were blemished with ethidium bromide and pictured on 2% agarose gel electrophoresis.

*Statistical analysis*

Statistical analyses were carried out using the SPSS software package 13.0 (SPSS Inc., Chicago, IL). Mean age for cases and controls was evaluated by using Student’s t-test. Odd ratios (ORs) and the corresponding 95% confidence intervals (CI) calculated by using logistic regression analysis were used to analyse the association between the polymorphic genotypes and CRC risk, based on heterozygous and homozygous comparison models with the wild type genotype served as the reference. Odds ratios of > 1.00 specified a positive risk association and vice versa. P values of < 0.05 were significant. 

## Results

*Study population*

The study was conducted through 200 patients of *RAD51* (*rs1801320*) and *XRCC2* *(rs3218536*) with a number of 200 controls. This study included 97 males (%) and 103 females (57.5%) colorectal cancer patients with an average age of 51 and the control group covered 87 males (43.5%) and 113 females (56.5%) with an average age of 47.

*RAD51 (rs1801320) polymorphism*

It was evident after gel electrophoresis that DNA fragment pattern of BstNI digestion of the PCR product was done to recognize the genetic variation at *RAD51 *(*rs1801320*). This population based case-control study was conducted to survey the prevalence of RAD51 polymorphism in Bangladeshi population of CRC patients and normal controls. The genotype frequencies in cases and controls are presented in Table 4. The C/C homozygous genotype was statistically lower in patients than in controls (63.24% vs. 73.77%).The frequency of G/C heterozygous genotype was found statistically significant (p=0.037; OR=1.64; 95% CI =1.03 to 2.60). The G/G genotype was not found statistically significant (p=0.423; OR=1.61; 95% CI = 0.49 to 5.22) and significance was observed for GC+GG (p=0.030; OR=1.63; 95% CI=1.05 to 2.55). Here, the risk factor of developing CRC is observed in G/C heterozygote and GC+GG (heterozygote + mutant) but no significance observed in case of G/G mutant.

*XRCC2 (rs3218536) polymorphism*

Digestion by Hph1 shows the mutation of *XRCC2 *(*rs3218536*). This population based case-control study was conducted to investigate the prevalence of *XRCC*2 polymorphism in Bangladeshi population of CRC patients and normal controls. The genotype frequencies in cases and controls are presented in Table 5. The C/C homozygous genotypes were statistically lower in patients than in controls (34.05% vs. 45.35%).The frequency of C/T heterozygous genotype was statistically significant (P=0.033; OR=1.60; 95% CI = 1.04 to 2.64). The T/T genotype was not found statistically significant (P=0.237; OR=1.65; 95% CI = 0.72 to 3.76) and significance was observed for CT+TT (p=0.027; OR=1.61; 95% CI=1.05 to 2.45).Here, the risk factor of developing CRC is observed in C/T heterozygote, CT+TT (heterozygote + mutant) and no significance observed in case of T/T mutant.

A comparison of previous association studies between *RAD51* (*rs1801320*) and CRC with present study is shown in Table 6 and similar comparative study between *XRCC2* (*rs3218536*) and CRC with present study is given in Table 7.

**Table 1:** Demographic Characteristics of the Patients and Controls

Variables	Cases n=185 (%)	Controls n=183 (%)
Age (Years)		
<45	95 (51.35%)	85 (46.45%)
45-60	67 (36.21%)	86 (46.99%)
>60	23 (12.43%)	12 (6.56%)
Sex		
Male	86 (46.48%)	77 (42.076%)
Female	99 (53.51%)	106 (57.92%)
Primary Tumor Location		
Colon	122 (65.95%)	N/A
Rectum	63 (34.05%)	N/A
Tumor Stage		
I	39 (21.08%)	N/A
II	85 (45.95%)	N/A
III	24 (12.97%)	N/A
IV	37 (20%)	N/A

**Table 2:** Correlation of* RAD51 *and *XRCC2* Polymorphism with Clinicopathological Characteristics of the Patients

Characteristics		rs1801320 Carrier (n=68)	rs1801320 Non carrier (n=117)	OR (95% CI)	*p*-value	rs3218536 Carrier (n=122)	rs3218536 Non carrier (n=63)	OR (95% CI)	*p*-value
Age (Years)									
<45	95 (51.35%)	31	64	0.69 (0.38-1.26)	0.237	67	28	1.52 (0.83-2.81)	0.178
45-60	67 (36.21%)	25	42	0.85 (0.44-1.63)	0.63	45	22	1.3 (0.67-2.53)	0.436
>60	23 (12.43%)	12	11	1.56 (0.62-3.91)	0.341	10	13	0.49 (0.19-1.23)	0.131
45-60 + >60		37	53	ref		55	35		
Sex									
Male	86 (46.48%)	32	54	ref		59	27	ref	
Female	99 (53.51%)	36	63	0.96 (0.53-1.76)	0.905	63	36	0.8 (0.43-1.48)	0.477
Primary Tumor Location									
Colon	122 (65.95%)	35	87	ref		95	27	ref	
Rectum	63 (34.05%)	33	30	1.7 (1.45-5.14)	0.002	27	36	0.21 (0.11-0.4112)	<0.0001
Tumor Stage									
I	39 (21.08%)	12	27			33	6	ref	
II	85 (45.95%)	37	48	1.73 (0.78-3.88)	0.179	47	38	0.22 (0.08-0.60)	0.002
III	24 (12.97%)	7	17	0.93 (0.30-2.82)	0.893	17	7	0.44 (0.13-1.52)	0.195
IV	37 (20%)	12	25	1.08 (0.41-2.84)	0.876	25	12	0.37 (0.12-1.15)	0.086

**Table 3:** Primers, PCR Conditions, Restriction Enzymes (RE) and Expected DNA Fragment on Digestion to Determine the Genotype

Allele	Primer Sequence	PCR Conditions	RE	DNA Fragments
*RAD51* (rs1801320)	FP:TGGGAACTGCAACTCATCTGG	95°C 30s	BstNI	GG: 71,86
	RP: GCGCTCCTCTCTCCAGCAG	56.5°C 30s		GC:71,86,157
		72°C 60s		CC:157
*XRCC2 *(rs3218536)	FP: TTGCTGCCATGCCTTACAGA	95°C 30s	Hph1	
	RP: TGGATAGACCGCGTCAATGG	52.1°C 30s		CC:234
		72°C 60s		CT:87,147,234
				TT:87,147

*FP, Forward Primer; RP, Reverse Primer; RE, Restriction Enzyme

**Table 4:** Genotype Frequencies of *RAD51* (rs1801320) Gene Polymorphism in Cases and Controls

Genotype	Cases N=185	Controls N=183	Odds Ratio	95% CI	*P-*Value
CC	117 (63.24%)	135 (73.77%)	---------	---------	---------
GC	61 (32.97%)	43 (23.49%)	1.64	1.03 to 2.6	0.037
GG	7 (3.78%)	5 (2.73%)	1.61	0.49 to 5.22	0.423
GC+GG	68 (36.75%)	48 (26.22%)	1.63	1.05 to 2.55	0.03

**Table 5:** Genotype Frequencies of *XRCC2* (rs3218536) Gene Polymorphism in Cases and Controls

Genotype	Cases N=185	Controls N=183	Odds Ratio	95% CI	*P*-Value
CC	63 (34.05%)	83 (45.35%)	---------	---------	---------
CT	107 (57.83%)	88 (48.08%)	1.6	1.04 to 2.46	0.033
TT	15 (8.11%)	12 (6.55%)	1.65	0.72 to 3.76	0.237
CT+TT	122 (65.94%)	100 (54.63%)	1.61	1.05 to 2.45	0.027

**Table 6:** Comparison of Previous Association Studies between *RAD51 *(rs1801320) and CRC with Present Study

Author	No. of Cases	C/C%	G/C%	G/G%	Association Status	Population
Krupa et al	100	61	36	3	Association↑	Poland
Romanowicz et al	100	66	18	16	Association↑	Poland
Cetinkunaret al	71	11	21	39	Association↑	Turkey
Nissaret al	100	19	56	25	Association↑	Kashmir
Yazdanpanahiet al	100	1	27	72	Association↓	Iran
Garstkaet al	52	8	33	11	Association↑	Poland
Present study	200	117	61	7	Association↑	Bangladesh

**Table 7:** Comparison of Previous Association Studies between *XRCC2* (rs3218536) and CRC with Present Study

Author	No. of Cases	C/C%	C/T%	T/T%	Association Status	Population
Krupaet al	100	75	18	7	Association↑	Poland
Present study	200	57	107	15	Association↑	Bangladesh

## Discussion

Protein haploinsufficiency regulation by genetic polymorphisms in homologous recombination repair (*HRR*) genes have an extremely immediate association with cancer threat. Both RAD51 and XRCC2 proteins are fundamental constituents of DNA double strand breaks (DSBs) reconstruction by HRR. Constructional at the same time functional correspondences are noted between *RAD51* and* XRCC*2 gene (Smilenov, 2006; Krupa et al., 2011). Genomic instability is demonstrated if cell deficient with any of *RAD51* or *XRCC2* genes products are defective in homologous recombination. The X-ray repair complementing defective repair in Chinese hamster cells 2 (*XRCC2*) gene plays an important role in the homologous recombination repair (HRR) pathway at the same time can be said a functional member for taking part in tumor progression (Griffin et al., 2000; Mohindra et al., 2002; Thacker and Zdzienicka, 2004). Improvement of unrepaired DNA destructions would be conducted by under expression of *RAD51* gene. Replication blunders past these destructions would guide to the growth of mutations and cancer.

Colon carcinoma can occur due to elevated-expression of *RAD51* and *XRCC2*. One of the significant mechanisms has been appeared by single nucleotide polymorphisms for apprehending the genes accountable for conferring susceptibility to cancer. Previously, studies related to *RAD51* and *XRCC2* genes polymorphisms have recognized association between the distribution of these polymorphisms and the vulnerability of colorectal adenoma or cancer. A Polish case-control study was outlined that polymorphism of the RAD51 can alter the colorectal cancer risk alone as well as with association with other polymorphisms: *XRCC2* gene (Krupa et al., 2011). Another Chinese case control study says that in CRC tissue, the expression of *XRCC2* is inflated and notable interdependences are also found among positive *XRCC2* expression, tumor size, Dukes’ stage and TNM stage (Xu et al., 2014). In case of Australian population, a comparative consequence of the *XRCC*2 alteration to cancer threat is remarked for members of this family (Park et al., 2012). Another case study on Polish population (Warsaw) also said that the feasible character of *XRCC2* gene polymorphism is responsible for neoplastic diseases. Therefore, *XRCC2* polymorphisms may take part dominant role in colorectal cancer tumorigenesis, conferring susceptibility to rectal tumors in Polish population (Romanowicz et al., 2016). Similar relationship is also observed when studied on English population (Cartwright et al., 1998). The investigation on *RAD51* and *XRCC2* gene polymorphism was done by being encouraged with strong association between these genes and these gene polymorphisms may be used in several areas in colon cancer management, such as early detection of the carcinogenesis, prediction of tumor biologic behaviour and being a candidate therapeutic target for colon cancer. Evidence is yielded by this study that *RAD51* and *XRCC2* gene polymorphisms may be risk factors for colon cancer progress (Cetinkunar et al., 2015). A result of meta-analysis says that the* RAD51 135 G/C* polymorphism is responsible for increased risk of CRC in total using allele (OR=1.21) and recessive models (OR=1.62). However, *XRCC2 rs3218536 A/G* was not associated with the risk of CRC in total or in subgroups in that particular study. As claimed by the consequences of meta-analysis, the* RAD51 135 G/C* polymorphisms might influence colorectal cancer risk (Eskandari et al., 2017). Few reports are also made out regarding the impact of *RAD51135G>C* in spread of CRC in Asian populations. In a subgroup of population of Kashmir, *RAD51135G>C* has marked the association with CRC (Nissar et al., 2014). On the other hand, in case of Iranian population *RAD51 135G>C* presumably does not impact on CRC susceptibility and other risk factors should be contemplated for furtherance of management potentials of the disease in Iran (Yazdanpanahi et al., 2018).

The outcomes found from our research are harmonious with various prior studies yet corresponding genotyping analysis in more racial communities is still necessary to confirm the association between SNPs and susceptibility to colorectal cancer. Our results confirmed that *RAD51 *(*rs1801320*) and *XRCC2 *(*rs3218536*) genes have increased probability of colorectal cancer and both genes are significantly associated with colorectal cancer occurrence and further elucidate the relationship of multiple genotypes at the *RAD51*(*rs1801320*) and *XRCC2 *(*rs3218536*) locus with colorectal cancer occurrence. 

In summary, studying SNP in *RAD51* (*rs1801320*) and *XRCC2* (*rs3218536*) genes helps to recognize the malfunction of these proteins and can lead to successful therapies for colorectal cancer. In this study, it is observed that the risk factor of developing CRC is noticed in G/C, C/T heterozygote and GC+GG, CT+TT (heterozygote+ mutant) in *RAD51* (*rs1801320*) and *XRCC2* (*rs3218536*) respectively but no significance is found in case of G/G and T/T mutant. So, it can be said that a significant association between *RAD51 *(*rs1801320*) and *XRCC2* (*rs3218536*) and colorectal cancer development is confirmed in our population. If the study can be conducted by using more samples from different areas, G/G and T/T mutant can also be found significant. However, it is the first study in Bangladesh and with a limited number of cases and controls. Therefore, *RAD51* *(rs1801320*) and *XRCC2 *(*rs3218536*) genes polymorphism and colorectal cancer risk is an important research area that needs much more attention as some statistically strong risk factors required to be excluded because of a small number of sample and control. It will also reduce the number of injuries or deaths resulting from incorrect or ineffective prescriptions prescribed for cancer patients and tend to integrate into personalized medicine.

## References

[B1] Cartwright R, Cathryn E, Paul J, Simpson, Thacker J (1998). The XRCC2 DNA repair gene from human and mouse encodes a novel member of the recA/RAD51 family. Nucleic Acids Res.

[B2] Cetinkunar S, Gok I, Celep RB, Ilhan D, Erdem H (2015). The effect of polymorphism in DNA repair genes RAD51 and XRCC2 in colorectal cancer in Turkish population. Int J Clin Exp Med.

[B3] Chang CJ, Hung MC (2012). The role of EZH2 in tumour progression. Br J Cancer.

[B4] Cook NR, Lee IM, Zhang SM, Moorthy MV, Buring JE (2013). Alternate-day, low-dose aspirin and cancer risk: long-term observational follow-up of a randomized trial. Ann Intern Med.

[B5] Cousineau I, Abaji C, Belmaaza A (2005). BRCA1 regulates RAD51 function in response to DNA damage and suppresses spontaneous sister chromatid replication slippage: implications for sister chromatid cohesion, genome stability, and carcinogenesis. Cancer Res.

[B6] Eskandari E, Rezaifar A, Hashemi M (2017). XPG Asp1104His, XRCC2 Rs3218536 A/G and RAD51 135G/C gene polymorphisms and colorectal cancer risk: A meta-analysis. Asian Pac J Cancer Prev.

[B7] Gildemeister OS, Sage JM, Knight Kl (2009). Cellular redistribution of RAD51 in response to DNA damage: novel role for RAD51C. J Biol Chem.

[B8] Griffin CS, Simpson PJ, Wilson CR, Thacker J (2000). Mammalian recombination-repair genes XRCC2and XRCC3 promote correct chromosome segregation. Nat Cell Biol.

[B9] Hussain SA, Sullivan R (2013). Cancer control in Bangladesh. Jpn J Clin Oncol.

[B10] Jackson TJ, Ahmed F, German RP, Lai SM, Friedman C (2006). Descriptive epidemiology of colorectal cancer in the United States, 1998-2001. Cancer.

[B11] Jemal A, Center MM, Desentis C, Ward EM (2010). Global patterns of cancer incidence and mortality rates and trends. Cancer Epidemiol Biomarkers Prev.

[B12] Krupa R, Sliwinski T, Wisniewska-Jarosinska M (2011). Polymorphisms in RAD51, XRCC2 and XRCC3 genes of the homologous recombination repair in colorectal cancer—a case control study. Mol Biol Rep.

[B13] Lin WY, Camp NJ, Cannon-Albright LA (2011). A role for XRCC2 gene polymorphisms in breast cancer risk and survival. J Med Genet.

[B14] Lynch HT, De’aChapelle A (2003). Hereditary colorectal cancer. N Engl J Med.

[B15] Mohindra A, Hays LE, Phillips EN (2002). Defects in homologous recombination repair in mismatch-repair-deficient tumour cell lines. Hum Mol Genet.

[B16] Neugut AI, Jacobson JS, Sherif G (1995). Coronary artery disease and colorectal neoplasia. Dis Colon Rectum.

[B17] Nissar S, Baba SM, Akhtar T (2014). RAD51 G135C gene polymorphism and risk of colorectal cancer in Kashmir. Eur J Cancer Prev.

[B18] Park DJ, Lesueur F, Nguyen-Dumont T (2012). Rare mutations in XRCC2 increase the risk of breast cancer. Am J Hum Genet.

[B19] Paulicova S, Chemelarova M, Petera J, Palicka V, Paulik A (2013). Hypermethylation of RAD51L3 and XRCC2 genes to predict late toxicity in chemoradiotherapy-treated cervical cancer patients. Folia Biol.

[B20] Romanowicz H, Brys M, Forma E, Smolarz B (2016). Lack of associations between the 4234G/C x-ray repair cross complementary 2 (XRCC2) gene polymorphism and the risk of endrometrial cancer among Polish population. J Gynaecol Res Obstet.

[B21] Santarell RL, Pierre F, Corpet DE (2008). Processed meat and colorectal cancer: a review of epidemiologic and experimental evidence. Nutr Cancer.

[B22] Shabnaz S, Ahmed MU, Islam MS (2015). Breast cancer risk in relation to TP53 codon 72 and CDH1 gene polymorphisms in the Bangladeshi women. Tumor Biol.

[B23] Smilenov LB (2006). Tumor development: haploinsufficiency and local network assembly. Cancer Lett.

[B24] Tariq K, Ghias K (2016). Colorectal cancer carcinogenesis: a review of mechanisms. Cancer Biol Med.

[B25] Thacker J, Zdzienicka MZ (2004). The XRCC genes: expanding roles in DNA double-strand break repair. DNA Repair (Amst).

[B26] Volkel P, Dupret B, Le Bourhis X, Angrand PO (2015). Diverse involvement of EZH2 in cancer epigenetics. Am J Trans Res.

[B27] Willett WC (200). Diet and cancer: an evolving picture. JAMA.

[B29] Xu K, Song X, Chen Z (2014). XRCC2 promotes colorectal cancer cell growth, regulates cell cycle progression and apoptosis. Med.

[B30] Yazdanpanahi N, Salehi R, Kamali S (2018). RAD51 135G>C polymorphism and risk of sporadic colorectal cancer in Iranian population. J Cancer Res Ther.

